# Surgical Treatment of Short Bowel Syndrome—The Past, the Present and the Future, a Descriptive Review of the Literature

**DOI:** 10.3390/children9071024

**Published:** 2022-07-10

**Authors:** Julian L. Muff, Filipp Sokolovski, Zarah Walsh-Korb, Rashikh A. Choudhury, James C. Y. Dunn, Stefan G. Holland-Cunz, Raphael N. Vuille-dit-Bille

**Affiliations:** 1 Department of Pediatric Surgery, Children’s University Hospital, 4056 Basel, Switzerland; julian.muff@unibas.ch (J.L.M.); stefan.holland-cunz@ukbb.ch (S.G.H.-C.); 2 Department of General and Visceral Surgery, Kepler University Hospital, 4020 Linz, Austria; filipp.sokolovski@kepleruniklinikum.at; 3 Department of Chemistry, University of Basel, 4058 Basel, Switzerland; zarah.korb@unibas.ch; 4 Department of Biosystems Science and Engineering, ETH Zurich, 4058 Basel, Switzerland; 5 Department of Transplant Surgery, University of Colorado Hospital, Aurora, CO 80045, USA; rashikh.choudhury@cuanschutz.edu; 6 Division of Pediatric Surgery, Stanford University School of Medicine, Stanford, CA 94304, USA; jdunn2@stanford.edu

**Keywords:** short bowel syndrome, SBS, STEP, LILT, Bianchi, serial transverse enteroplasty procedure, longitudinal intestinal lengthening and tailoring, intestinal transplantation

## Abstract

Short bowel syndrome (SBS) is a devastating disorder with both short- and long-term implications for patients. Unfortunately, the prevalence of SBS has doubled over the past 40 years. Broadly speaking, the etiology of SBS can be categorized as congenital or secondary, the latter typically due to extensive small bowel resection following diseases of the small intestine, e.g., necrotizing enterocolitis, Hirschsprung’s disease or intestinal atresia. As of yet, no cure exists, thus, conservative treatment, primarily parenteral nutrition (PN), is the first-line therapy. In some cases, weaning from PN is not possible and operative therapy is required. The invention of the longitudinal intestinal lengthening and tailoring (LILT or Bianchi) procedure in 1980 was a major step forward in patient care and spawned further techniques that continue to improve lives for patients with severe SBS (e.g., double barrel enteroplasty, serial transverse enteroplasty, etc.). With this review, we aim to provide an overview of the clinical implications of SBS, common conservative therapies and the development of operative techniques over the past six decades. We also provide a short outlook on the future of operative techniques, specifically with respect to regenerative medicine.

## 1. Introduction

### 1.1. Definitions

Short bowel syndrome (SBS) in children is defined as “the need for parenteral nutrition (PN) for >60 days after intestinal resection or a bowel length of less than 25% of expected” by the North American Society for Pediatric Gastroenterology, Hepatology and Nutrition (NASPGHAN) [1]. SBS reflects the most common cause for intestinal failure (IF), which is defined by the European Society for Clinical Nutrition and Metabolism (ESPEN) as “the reduction of gut function below the minimum necessary for the absorption of macronutrients and/or water and electrolytes, such that intravenous supplementation is required to maintain health and/or growth” [2]. Other causes of IF include intestinal dysmotility, intestinal fistula, mechanical obstruction and extensive small bowel mucosal disease [2]. SBS remains a clinical diagnosis. In adults, SBS has typically been diagnosed when a patient has a total functional small bowel length of less than 200 cm with requirement for nutritional and/or fluid substitution [3]. In children, a clear definition of short bowel in terms of length in absolute numbers has not been made, likely due to the fact that pediatric intestinal length is linked to the state of growth [2]. However, in 1967 Rickham was the first to attempt to define short bowel as a remaining intestine of 30% of normal length, or in absolute numbers less than 75 cm, in a full-term neonate [4]. In children, SBS can be further categorized into: “very SBS” (≤40 cm), “ultra SBS” (between <30 cm and <10 cm) and “no gut syndrome” (only duodenum is left) [5,6].

**Figure 1:**
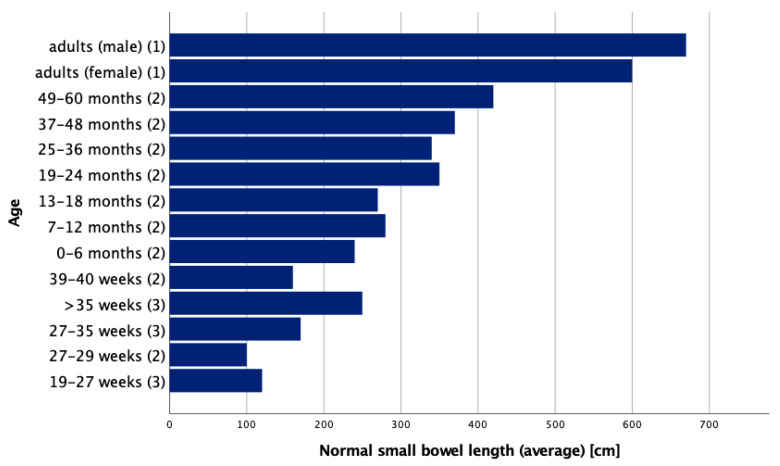
Normal small bowel lengths in various ages adapted from (**1**) Hounnou et al.; (**2**) Struijs et al.; (**3**) Touloukian et al. [8,9,10].

The length of the small intestine of adults measured from the ligament of Treitz to the ileocecal valve ranges from about 275 cm to 850 cm [3], with an average of about 360 cm [7]. Another study based on human adult cadavers reports average small intestinal lengths as 670 cm in males and 599 cm in females [8]. According to a cadaver study by Touloukian and Smith in term infants, the average small intestinal length is reported to be around 250 cm. During fetal development, the small intestine measures approximately 120 cm in the second trimester and doubles during the third trimester [9]. Another more recent study by Strujis et al. in regard to small bowel length measured in vivo states with typical lengths of 100 cm at a gestational age of 27–29 weeks, 160 cm at 39–40 weeks and 240 cm between zero and six months of age, and they showed that small bowel length is best correlated with body length [10]. An overview of average small bowel lengths in various (gestational) age groups is provided in Figure 1. In general, there are great inconsistencies in the description of normal small bowel lengths in the literature.

SBS may result from congenital or acquired conditions. Mostly, SBS results from extensive surgical resection of the small intestine due to midgut volvulus, malrotation, extensive aganglionosis or necrotizing enterocolitis [11], the latter reflecting the most common cause of bowel resection in children (29% of cases) [12]. In adults, multiple small bowel resections related to recurrent Crohn’s disease or massive resections due to catastrophic mesenteric events (arterial embolism, venous thrombosis), trauma or malignancies represent the most common causes of SBS [11,13]. A comprehensive overview of causes of small intestinal resection in children and adults is provided in Table 1.

The incidence of SBS is reported by a Canadian study as 24.5 per 100,000 live births, and is much greater in premature births (353.7 per 100,000) [14]. Exact data on the prevalence of SBS do not exist. Data on home parenteral nutrition (HPN) are used as a proxy to estimate the prevalence of SBS. An European multicenter study from 1999 reports a point prevalence of four per million inhabitants, 35% of which received HPN because of SBS [15]. Another study reports the annual prevalence of HPN in the United States as one-hundred and twenty per million, approximately a quarter receiving HPN due to SBS [16]. DiBaise et al. state that these numbers seem to be an underestimation of the real SBS prevalence, as they do not reflect SBS patients who either did not require PN or were able to be successfully weaned from PN. As reasons for the differences between the US and Europe, they quote that the rate of HPN in the US might be higher, as patients may be discharged from the hospital sooner on home PN due to financial considerations, as well as that the organization of HPN resources in the US is well established and available [13].

### 1.2. Clinical Implications of SBS

SBS is associated with significant fluid and electrolyte losses and decreased ability to absorb macronutrients (i.e., carbohydrates, fat and protein), vitamins and minerals [17]. Clinical symptoms of SBS include diarrhea, steatorrhea, malnutrition, dehydration and, ultimately, failure to thrive [2]. In the past, SBS was associated with a high mortality rate. Advances in medical care, including the use of PN, has improved survival and quality of life, and generally improved the prognosis of children affected by SBS in developed countries. The goal of treatment is to increase the absorption capacity of the remaining bowel in order to achieve enteral autonomy [2]. Enteral autonomy is defined by the American Society for Parenteral and Enteral Nutrition (ASPEN) as “the maintenance of normal growth and hydration status by means of enteral support without the use of parenteral support for a period of more than three consecutive months” [18].

#### 1.2.1. Proximal Versus Distal Small Bowel Loss

Patients affected by proximal resections (i.e., jejunum) tend to have a transitory but significant hypersecretion of gastric acid lasting a few weeks to months. This is likely due to unopposed gastric acid secretion by decreased production of endogenous inhibitors such as secretin, cholecystokinin, neurotensin and vasoactive intestinal peptide. Gastric hypersecretion results in inactivation of pancreatic enzymes, aggravation of diarrhea, precipitation of peptic ulcer disease and worsened malabsorption [19,20]. Nonetheless, patients with proximal resections seem to have better postoperative bowel function than those with distal resections (i.e., ileum). The ileum has significant fluid and electrolyte absorptive capacity and is the main site of bile acid and vitamin B12 absorption [17,21]. Additionally, reduced production of intestinal mediators peptide YY (PYY) and glucagon-like-peptide 1 (GLP-1), which all are produced in the ileum and colon and physiologically inhibit intestinal motility, results in increased intestinal transit time. Glucagon-like-peptide 2 (GLP-2) is also produced in the ileum and the colon and increases nutrient absorption, maintains mucosal integrity, and enhances small and large intestinal villus and crypt cell growth; therefore, these beneficial effects are diminished in distal resections [19].

Expression of nutrient (i.e., amino acid, peptide and monosaccharide) transporters along the small intestine is asymmetric, with some transporters being expressed to a greater extent in the jejunum and others in the ileum [22,23,24,25]. Furthermore, transporter expression does not necessarily reflect absorption. While some transporters have been shown to functionally interact [26,27,28,29], others depend on the presence of certain accessory proteins (e.g., angiotensin converting enzyme 2 (ACE2)) [22,25,30]. Expression of some transport proteins such as GLUT2 and SGLT1 is comparable between the newborn and adult small intestine. This extends to the expression of mRNA of the transporters GLUT1, GLUT2, GLUT7 and SGLT1. GLUT5, on the other hand, is not expressed in the newborn human gut on a protein level, but its mRNA is expressed similarly as in adults [24].

#### 1.2.2. Choleretic Diarrhea

Increased amounts of bile acids entering the colon result in elevated bacterial deconjugation to free bile acids and subsequent stimulation of chloride and water secretion, as well as intestinal motility, a condition referred to as choleretic diarrhea [20]. A disrupted enterohepatic circulation, which manifests as diminished bile acid reabsorption and subsequent supersaturation of cholesterol in bile, alone or in conjunction with bile stasis due to little to no enteral stimulation in patients on PN, may lead to cholelithiasis. This condition occurs in 25–45% of SBS patients [19].

#### 1.2.3. Small Intestinal Bacterial Overgrowth

Another significant issue in SBS is small intestinal bacterial overgrowth (SIBO). Following massive resection, the remaining bowel undergoes intestinal adaptation in order to increase fluid and nutrient absorption to compensate for lost bowel. This manifests as structural and functional changes, i.e., increase in absorptive capacity due to bowel dilatation and decrease in bowel transit time [31]. Furthermore, enterocytic hyperplasia, increased villous height and crypt depth, and increased expression of transporter proteins occur [32]. This process starts shortly after surgery and continues for up to 60 months, being most active in the first two years [1]. Both altered intestinal motility and bacterial translocation, as occurs in cases of ileocecal valve (ICV) resection, act as risk factors for SIBO [17]. Additionally, some medications such as antimotility agents or acid-suppressing drugs have the potential to disrupt native physiological flora and stimulate SIBO [20]. SIBO may result in mucosal inflammation, either due to direct cytopathic effects of bacteria or inappropriate immune reactions to absorbed bacterial antigens [31]. The consequences of mucosal inflammation may manifest as epithelial changes; for example, blunting of villi [33], less visible damage to the brush border membrane and/or production of inflammatory mediators/cytokines, all of which impair absorption [31]. Intraluminal bacterial degradation of carbohydrates leads to production of carbon dioxide, methane or hydrogen. Intraluminal deconjugation of bile acids results in insufficient concentrations, leading to fat malabsorption [31]. In this setting, the clinical symptoms of SIBO include gas, bloating, abdominal discomfort, diarrhea, steatorrhea, oxalate kidney stones and symptoms of deficiencies of fat-soluble vitamins A, D and E, but rarely vitamin K, as enteric bacteria are able to produce some amounts of vitamin K and folate [31]. Impaired vitamin B12 absorption, due to bacterial sequestration, may lead to macrocytic anemia and neurologic symptoms [31]. Intestinal inflammation can also manifest in anastomotic ulcerations resulting in chronic bleeding and microcytic anemia [31]. Another rare complication in children with SBS is D-lactic acidosis, which occurs due to degradation of excess carbohydrates by lactobacillus bacteria producing D-lactic acid. Symptoms include acidosis, lethargy and confusion [17]. In addition to the aforementioned symptoms and conditions, other potential complications of SIBO include recurring bacterial infections and sepsis, failure to thrive and delayed weaning from PN [31]. Duodenal aspirate and culture are considered the gold standard in the diagnosis of SIBO, with cut-offs defined as >10^3^ colony forming units (CFU) coliforms/mL [34]. However, this technique has several limitations, as it is costly and invasive due to the requirement of upper endoscopy. In addition, it is prone to contamination by upper gastrointestinal flora which may yield false-positive results, and since mid and distal segments of the small intestine are beyond the reach of regular endoscopes, cultures from duodenal aspirates may be false-negative [35,36]. Another possible way of diagnosing SIBO is measurement of exhaled hydrogen upon ingestion of either 75 g of glucose or 10 g of lactulose. A rise in exhaled hydrogen of at least 20 parts per million (ppm) compared to baseline 90 min after oral ingestion is diagnostic for SIBO [34]. Although breath tests are used more frequently, they lack universal acceptance [35]. A recent comparative study by Cangemi et al. comparing duodenal aspirate (DA) to the lactulose breath test (LBT) showed that contamination is frequent in DA (19.8%) and there is poor result agreement between DA and LBT (63.5%). Therefore, the authors recommend the use of LBT as it is cheaper, safer, and less likely to yield a contaminant result [37].

## 2. Conservative Treatment

The ultimate goal of SBS treatment is to achieve full intestinal autonomy and to reduce long-term dependence on parenteral support by increasing the absorptive capacity of the remnant bowel [17]. Current therapeutic approaches follow a sequential strategy. The first-line therapy consists of parenteral nutrition, promotion of enteral feeding, restoration of bowel continuity, closure of enterostomies as early as possible, the use of dietary supplementations and, where indicated, antibiotics [38]. In some cases, intestinal growth can be stimulated by the use of growth factors [38].

### 2.1. Parenteral Nutrition

Since its advent in the late 1960s [39], PN represents a cornerstone in the conservative treatment of SBS patients. PN aims to replete calories and nutrients while bypassing the enteral circuit [17]. The European Society of Paediatric Gastroenterology, Hepatology and Nutrition (ESPGHAN), European Society for Clinical Nutrition and Metabolism (ESPEN), European Society of Paediatric Research (ESPR) and Chinese Society of Parenteral and Enteral Nutrition (CSPEN) provides recommendations in its 2018 published guideline on the use of pediatric parenteral nutrition [40]. The guideline recommends the use of Schofield’s equation for calculation of resting energy expenditure (REE), which is based on age, weight and gender; additionally, energy requirements for preterm and term newborns and infants for PN in different stages of disease are provided [41]. Exact parenteral glucose supply recommendations for preterm and term newborns, as well as infants and children, are provided, the latter according to their body weight and phase of illness. Hyperglycemia (>145 mg/dL) should be avoided in pediatric and neonatal ICU patients due to increased morbidity and mortality. In addition, repetitive and/or prolonged hypoglycemia (<45 mg/dL) should be avoided in all ICU patients. Excessive glucose intake should be avoided, as hyperglycemia causes liver steatosis and enhanced hepatic VLDL production [42]. Parenteral amino acid intake in preterm newborns should be at least 1.5 g/kg/d on the first postnatal day and between 2.5 g/kg/d and 3.5 g/kg/d from postnatal day two onwards, alongside non-protein intakes >65 kcal/kg/d and adequate micronutrient intake. Term infants should receive between 1.5 g/kg/d and 3.0 g/kg/d of amino acids, and children and adolescents between 1.0 g/kg/d and 2.0 g/kg/d [43]. The first choice of treatment should be 20% intravenous lipid emulsions (ILEs). Parenteral lipid intake should be limited to 4 g/kg/d in preterm and term infants, respectively, and 3 g/kg/d in children, while markers of liver integrity and function, alongside triglyceride concentrations, should be monitored regularly in patients receiving ILEs. Consideration should be given to reducing ILE dosage if serum or plasma triglyceride concentration exceeds 265 mg/dL in infants or 400 mg/dL in older children [44]. Infants and children on PN should receive parenteral vitamins daily, except for vitamin K which should be given weekly [45]. Newborns and children requiring prolonged PN during hospitalization should receive either a peripherally inserted central catheter (PICC) or a tunneled central vein catheter (CVC); for long-term or home PN children a tunneled CVC is the preferred choice. Where possible, a CVC should only be used for administering PN. Taurolidine is effective in preventing catheter related bloodstream infections (CRBSI) and should be used during long-term catheter use. Use of heparin flush over saline flush to prevent thrombotic occlusion has no proven benefit in children and is, therefore, not recommended [46]. Standard PN solutions, over individualized PN solutions, should be used in the majority of pediatric and newborn patients including very-low-birthweight premature infants [47].

The ESPGHAN/ESPEN/ESPR/CSPEN guidelines also provide recommendations on complications [48], fluid and electrolytes [49], iron and trace minerals [50], calcium, phosphorus and magnesium [51], home parenteral nutrition [52] and organizational aspects [53] in PN. A study by Gonzalez-Hernandez et al. on clinical variables predicting time to weaning from PN showed that time to full enteral nutrition is strongly associated with both the remaining small bowel length and the percentage of expected remaining small bowel length based on gestational age (GA). Children who have 26–50% of expected small bowel remaining required PN for approximately two years, compared to children with 51–75% remaining of expected small bowel who required PN for approximately one year. Factors that showed no difference in time to full enteral nutrition were gender, birth weight, GA, underlying diagnosis requiring intestinal resection, presence or absence of the ICV and type of anastomosis (small bowel to small bowel anastomosis vs. small bowel to colon anastomosis) [54].

### 2.2. Medical Management

The focus of medical treatment is to compensate for fluid, electrolyte and nutrient losses, to limit diarrhea and promote adequate weight gain and growth [17].

#### 2.2.1. Antimotility Agents

The first-line therapy for diarrhea is antimotility agents such as loperamide or diphenoxylate-atropine [20]. Loperamide is a peripherally acting µ-opioid receptor agonist and is not associated with undesirable central nervous system (CNS) effects, such as sedation or addiction [20]. In contrast to loperamide, the opioid receptor agonist diphenoxylate crosses the blood–brain barrier [55]. Nevertheless, the abuse potential of diphenoxylate is limited, as atropine causes anticholinergic side effects such as xerostomia, tachycardia or mydriasis when taken in high doses [55]. Other second- or third-line treatments are opioids such as codeine, morphine or opium tincture, which are not restricted to the peripheral nervous system and can induce significant CNS effects [56]. Unlike tolerance to analgesic effects, tolerance to antidiarrheal effects of opioids and opioid-receptor agonists are rare [20,57].

#### 2.2.2. Choleretic Diarrhea Treatment

Patients with choleretic diarrhea can be treated with bile-acid binding resins such as cholestyramine, colestipol or colesevelam. These are non-digestible anion exchange polymers that bind to bile acids in the colon and form insoluble complexes, which are then excreted [20]. In patients with extensive ileal resection, bile-acid binding resins may not be appropriate, because the loss of bile acids might be greater than the hepatic synthesis, and treatment with resins may aggravate fat malabsorption and steatorrhea [20]. Furthermore, resins should be used with caution because of possible interactions (i.e., binding and reducing activity) with other drugs such as loperamide or nonsteroidal anti-inflammatory drugs [20].

#### 2.2.3. Inhibition of Gastric Hypersecretion

Gastric hypersecretion can be addressed with several drug classes. Proton pump inhibitors (PPI) such as pantoprazole, omeprazole, lansoprazole or esomeprazole, which irreversibly block the H^+^-K^+^-ATPase proton pump, are the first-line treatment and show great efficacy [20]. Although generally well tolerated, long-term use of PPIs increases the risk for osteoporosis, bone fractures and vitamin B12 deficiency [58,59]. Upon discontinuation, rebound hypersecretion may occur [60]. As gastric acid physiologically reduces the concentration of ingested bacteria, PPI treatment may stimulate SIBO [61]. Histamine type 2 (H2) receptor antagonists (e.g., famotidine, ranitidine or cimetidine) block the action of the acid secretion mediator histamine, which is released from gastric mucosa upon stimulation by gastrin. They act as second-line agents along with a_2_-adrenergic receptor agonists, such as clonidine, although evidence for use of the latter is scant [20]. Third-line treatment includes the lone-acting somatostatin analogue octreotide, which inhibits gastrin and other GI hormones [20]. The use of octreotide has several limitations, including high cost, inconvenient administration (subcutaneous injection) and possible risk of adverse effects such as cholelithiasis [20].

#### 2.2.4. Treatment of Small Intestinal Bacterial Overgrowth

The American College of Gastroenterologists (ACG) recommends antibiotic treatment for SIBO in order to eradicate overgrowth and resolve symptoms, citing a meta-analysis of 24 cohort studies, 7 randomized controlled trials (RCTs) and 1 randomized crossover study showing rifaximin is effective and safe for treating SIBO [34,62]. Other antibiotics such as amoxicillin-clavulanic acid, ciprofloxacin, norfloxacin, doxycycline, metronidazole, neomycin, tetracycline and trimethoprim-sulfamethoxazole are also suggested in the ACG guideline, with variable efficacies in eradicating SIBO ranging from 30% to 100%; in general, evidence is limited to small clinical studies of poor to modest quality [34]. A meta-analysis of twelve RCTs, two crossover trials, four prospective single-arm trials and four retrospective studies on the use of probiotics in SIBO showed that probiotics are effective in terms of SIBO decontamination, reduction in H2 concentration and abdominal pain relief, but not in prevention of SIBO. The majority of included studies (21/22) focused on adult patients [63]. Furthermore, a systematic review of one crossover RCT, one case control study and nine case reports on the use of probiotics in children with SBS showed insufficient evidence [64].

#### 2.2.5. Glucagon-Like Peptide-2

A subcutaneously administered glucagon-like peptide-2 (GLP-2) analogue (teduglutide) has been found to increase intestinal absorption and reduce the need for parenteral support. It is approved in the European Union for SBS patients older than one year who are stable after a period of postoperative intestinal adaptation. Dosage is 0.05 mg/kg/day for both children and adults, and contraindications are active or suspected malignancy and/or a recent (i.e., ≤5 years) history of gastrointestinal (GI) or hepatobiliary malignancy. Teduglutide is generally well tolerated, but the most common adverse effects are abdominal distention and GI stoma complications (swelling of the stoma, etc.), each occurring in approximately 20% of patients [65]. One of the most important studies on the efficacy and safety of teduglutide in pediatric patients with intestinal failure due to SBS was a 24-week phase III study by Kocoshis et al.; 59 patients aged 1–17 years were randomized into either the 0.025 mg/kg/d (*n* = 24) or the 0.05 mg/kg/d (*n* = 26) or the standard of care (SOC) group (*n* = 9). Primary endpoint was a ≥20% reduction in parenteral support from baseline in week 24, which was achieved by 54.2% (*n* = 13) in the 0.025 mg/kg/d group, 69.2% (*n* = 18) in the 0.05 mg/kg/d group and 11.1% (*n* = 1) in the SOC group (*p* < 0.05 vs. SOC). In total, five patients, two receiving 0.025 mg/kg/d (8%) and three receiving 0.05 mg/kg/d (12%), but no patient in the SOC group, achieved enteral autonomy [66].

#### 2.2.6. Pancreatic Enzyme Replacement and Bile Acid Supplementation

Fat malabsorption can be treated with pancreatic enzyme replacement (i.e., pancrelipase). In order to maximize effectiveness, therapy should only be started following the normalization of gastric hypersecretion and GI motility with acid secretion and antimotility drugs. Bile acid supplementation may be beneficial to patients who cannot synthesize enough to compensate for losses. However, this should be used with caution, as exacerbation of diarrhea constitutes a possible side effect. Both pancreatic enzymes and bile acids should be taken with meals [20]. However, there are no data available on the use of bile acid supplementation in children with SBS.

#### 2.2.7. Chyme Reinfusion

An additional possible conservative treatment for intestinal failure in cases where patients have double enterostomies or entero-atmospheric fistulas is chyme reinfusion (CR). Here, upstream chyme is sucked and pumped into a tube inserted into the downstream intestine by a peristaltic pump in order to artificially restore bowel continuity pending surgical closure [67]. It is recommended by the American Society for Parenteral and Enteral Nutrition (ASPEN) [68] and the ESPEN [69,70,71] whenever possible. CR is very effective in restoring intestinal function, reducing the need for intravenous support by 89% [67]. CR is also effective in neonatal and pediatric populations, but available data are limited [72]. A study by Gause et al. on mucous fistula refeeding in preterm neonates after bowel resection and small bowel enterostomy demonstrated that, in the inter-operative period, CR patients reached goal enteral feedings and were able to have PN discontinued significantly earlier than patients with an enterostomy only, without creation of a mucous fistula and refeeding efforts. Moreover, after anastomosis, refeeding patients reached goal enteral feeds and had PN discontinued significantly earlier [73].

## 3. Surgical Treatment

### 3.1. Indications

Unlike as for intestinal transplantation [74], clear guidelines on indications for non-transplant surgery for SBS do not yet exist. The role and timing of non-transplant surgery for SBS patients remains a matter of debate, with some authors claiming it should be reserved solely for patients unable to wean from PN after unsuccessful use of all available medical measures [75]. According to some clinicians, surgery is only indicated if the patient has reached a plateau in bowel adaptation, and definitely requires long-term PN. In this view, as long as enteral feeding progresses, surgery is not warranted. Since the adaptation process is highly variable and may take years, it is advisable to wait a sufficient period of time to facilitate adaptation and attempts to increase enteral intake by conservative treatment [38]. The same authors state that surgery is indicated in patients who are unable to achieve at least 10% to 50% of caloric requirements enterally after six months of adequate conservative treatment [38]. Others state that bowel lengthening procedures should be indicated in patients on long-term PN only with substantial bowel dilatation. Most authors suggest lengthening surgery for patients reaching the plateau of intestinal adaptation or when complications relating to repeated episodes of bacterial overgrowth occur [76]. From a principle point of view, targets of non-transplant surgery for SBS are small bowel dilatation, rapid intestinal transit time and short remaining proximal small bowel, these being the most important reasons for failure of weaning off PN [77]. End-stage liver disease is a recognized contraindication for bowel lengthening surgical procedures in SBS patients, as these patients seem to benefit more from transplantation [38,78].

The ESPEN recommends, with regard to non-transplant surgery in patients with SBS, that bowel length should be conserved maximally during bowel resection, intestinal continuity should be achieved whenever possible and bowel lengthening procedures should be considered in selected patients. Clear recommendations for the timing and choice of procedure do not exist and are more related to the surgeon’s preference [74].

### 3.2. Procedures Increasing Small Intestinal Length

The main aim of these procedures is to increase the small intestinal length in order to expand the nutrient absorbing capacity of the guts. However, this is not the only consequence. All the procedures discussed in this chapter also result in a decreased diameter of the small intestine, thus reducing the risk of small intestinal bacterial overgrowth as discussed in Section 1.2.3. Furthermore, intestinal motility can be improved, leading to a reduction in stasis, also reducing the risk of bacterial overgrowth [17,31]. All these changes subsequently lead to a decreased need for parenteral nutrition.

#### 3.2.1. Serial Transverse Enteroplasty Procedure (STEP)

The serial transverse enteroplasty (STEP) procedure was first described by Kim et al. in 2003 in an animal model using young pigs [79]. In the same year, Kim et al. reported the first case of a STEP in humans, where they used the technique in a 2-year-old boy born with gastroschisis and midgut volvulus. He was previously unsuccessfully treated with LILT [80]. A first study by the same group in 2005 reporting on the short-term-outcomes of five pediatric patients showed an average increase in small bowel length of 82% (34–132%) [81]. STEP is currently considered to be the most common small bowel lengthening procedure worldwide [82].

**Figure 2:**
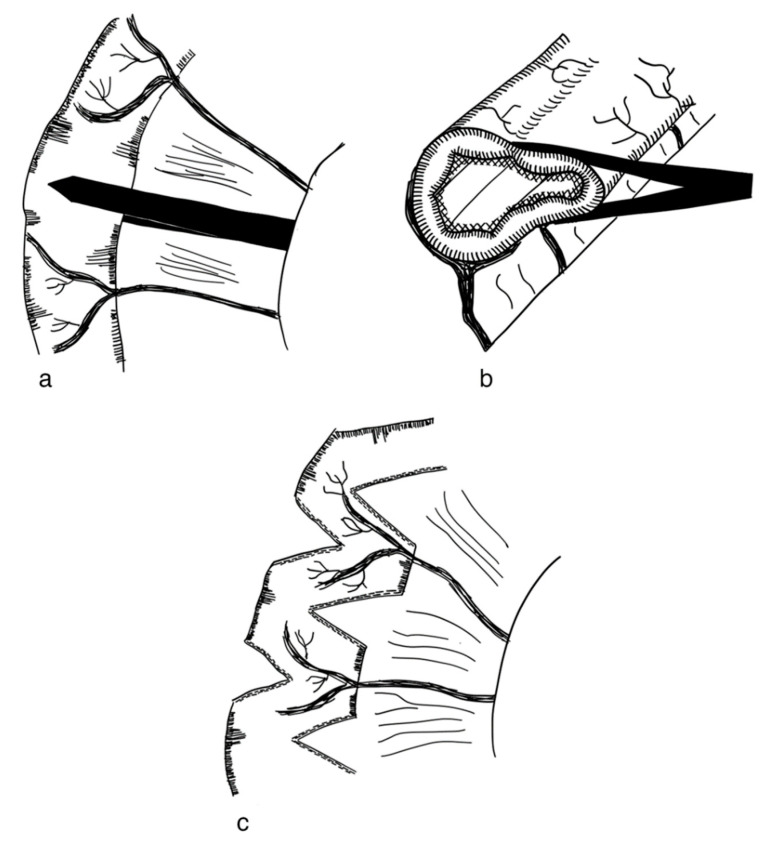
(**a**) With alternating cuts along the small intestine using a stapler (**b**) without damaging the mesentery (**c**), a zig-zag pattern is achieved, increasing the length of the small intestine. Kim et al. [79].

The STEP procedure uses alternating cuts with a stapler along the small intestine. Through this, a zig-zag pattern is achieved, leading to a decrease in bowel width and a subsequent increase in length [79]. Figure 2 illustrates the STEP procedure. The effect of multiple transverse cuts on the enteric nervous system and the smooth muscle complex has not been well studied.

Studies report age at operation ranging from the first day of life [83] up to the age of 14 years [84]. Multiple studies report successful STEP procedures performed in neonates [81,83,85]. However, in the neonatal period STEP should be limited to patients with severely short and dilated bowels [86]. It has been reported that STEP can be performed at any length of the dilated small intestine [82,87]; however, a minimum of 3.5–4 cm of diameter is required [82].

A second STEP procedure in children presenting with bowel dilation and an unsuccessful attempt at weaning off PN after the initial STEP is believed to add further benefit to most children [88]. Postoperative outcomes after reSTEP and single STEP are similar, supporting the use of reSTEP when clinically indicated [89]. However, reSTEP in young infants with gastroschisis may present a higher risk of failure due to intrinsic bowel dysmotility [89]. Reasons for reSTEP may comprise persistent inflammation and impaired mucosal growth, leading to continuing bowel dysfunction [90]. A study by Barrett et al. comparing first STEP with reSTEP concluded that reSTEP patients did not achieve enteral autonomy, and reSTEP should therefore be considered carefully [91]. 

A study by Frongia et al. showed that a simultaneous STEP and small bowel transplantation in porcine models due to size mismatch is technically feasible [92].

STEP comprises the risks of staple line leaks, sepsis, obstructions, bleedings, intestinal re-dilatation and abdominal abscess formation [93,94]. Summarizing the limited evidence (comprising retrospective case series and being highly prone to bias), the median percentage of bowel lengthening following STEP is around 49% and approx. 45% of patients are fully weaned from PN after STEP [95]. STEP is considered less difficult than LILT [82]. The reported mortality in 318 summarized patients was 7% for STEP [95].

A recently published Markov decision (with a level of evidence of III) reports that STEP has positive effects on enteral autonomy and long-term survival, as well as a reduction in the need for intestinal transplantation [96]. One of the most recent published studies in 36 patients operated on using STEP reports a median increase in bowel length of 53%, and 42% of patients regaining enteral autonomy [94]. Since 2005, Bueno et al. uses a modified version of the STEP. The MSTEP procedure applies the stapler in the same fashion as in the STEP procedure, however, without creating mesenteric defects while inserting the stapler arm. This technique can also be applied to the duodenum. The inventors reported this surgery to be at least as effective as the original STEP, with 5/11 (45%) patients achieving enteral autonomy [97]. Other modifications also report the possibility to lengthen the duodenum by the sequential transverse anterior and posterior application of an endoscopic stapler [98] (level of evidence: case series).

#### 3.2.2. Longitudinal Intestinal Lengthening and Tailoring Procedure (Bianchi Procedure)

**Figure 3:**
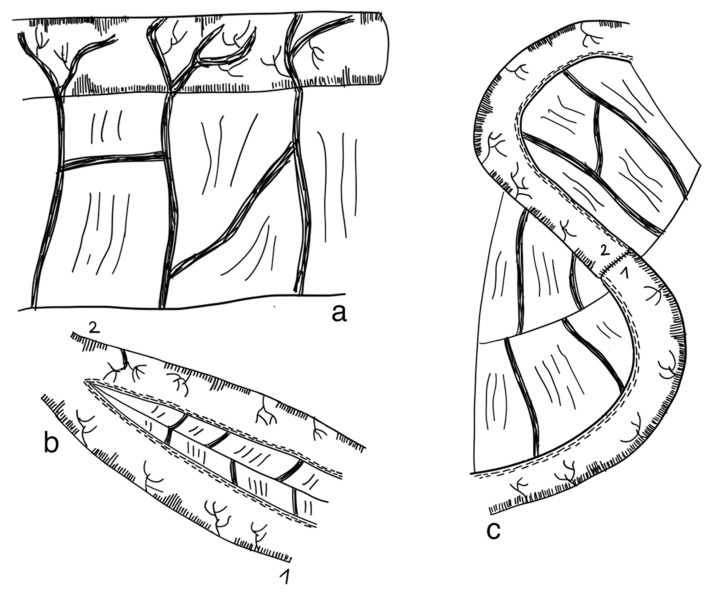
(**a**) A section of the dilated small intestine (view from the side) (**b**) is divided longitudinally into two intestinal halves (view from above). (**c**) These parts are then fashioned together into two narrower tubes. Numbers 1–2 illustrate the movement and new placement of the two intestinal halves. Bianchi [100].

Longitudinal intestinal lengthening and tailoring (LILT) was first introduced by Bianchi in 1980 as ‘Intestinal loop lengthening’ using a porcine model [99]. Hereby, a section of the dilated small intestine is divided longitudinally into two intestinal halves. These parts are then fashioned together into two narrower tubes, doubling the length of the original intestinal segment [100] (Figure 3). The LILT procedure is based on the observation that mesenteric vessels split before reaching the bowel wall, representing a posterior and anterior branch [101]. Thus, a safe division into two longitudinal halves with adequate blood supply can be performed [102]. The first case series was published in 1997 reporting 20 successful operations in children between 1982 and 1997. No operative mortality was reported. After more than six years follow-up, overall survival was 45% [103]. In 2006, Bianchi proposed preoperative controlled bowel expansion by intermittent occlusion of the jejunum with a large tube, increasing the length of small intestine suitable for lengthening procedures [104].

In order to perform a LILT procedure successfully, the small bowel should be dilated at least 200% compared to its normal diameter and should have a minimum length of 20–40 cm [38,103,105,106]. Second, an elastic mesentery should be present in order to avoid tension on the vasculature [101,102,103]. In the neonatal period and in infants up to 6 months of age, the LILT procedure remains controversial. Some authors state that LILT should be avoided during this time [105,107], as severe complications and unsatisfactory results have been observed [38], while others report that LILT is possible during the neonatal period [103,108]. Furthermore, LILT should not be performed as a secondary bowel lengthening procedure [82]. However, in a case report, LILT was successfully performed after an initial STEP procedure [109]. 

LILT involves the risks of anastomotic leakage, sepsis, re-dilatation, strictures, adhesions, perforations and fistulizations [38,75,82]. One of the most feared complications is intestinal necrosis. However, this has not been reported in the recent literature [82].

During the first postoperative year, complications due to the LILT procedure are reported to be more prevalent, e.g., several anastomoses are needed with the risk of leakage [38,106,107,110,111]. 

Evidence on the efficacy and safety of LILT is limited to case series and is highly prone to (publication) bias [95]. Summarizing a systematic review by Nagelkerke et al., 169 of 324 (52%) patients were fully weaned from PN following LILT [95], while survival rates of SBS patients evaluated for intestinal transplantation who underwent LILT compared to patients who did not were not significantly different [107]. Mortality of LILT was reported to be 26% [95]. One of the most recent studies by Shah et al. reported 5/9 patients operated on with LILT were weaned off parenteral nutrition [112]. Most studies evaluating the LILT procedure have, however, been published before the year 2010, indicating that these days the LILT procedure has been replaced in most institutions [103,106,107,110,111,113,114,115,116,117,118,119,120,121]. In addition to its potential risks, the LILT procedure has been described as technically very challenging, possibly contributing to the decline in execution and publication of LILT [82] (level of evidence: case series).

#### 3.2.3. Modification of LILT with One Anastomosis

**Figure 4:**
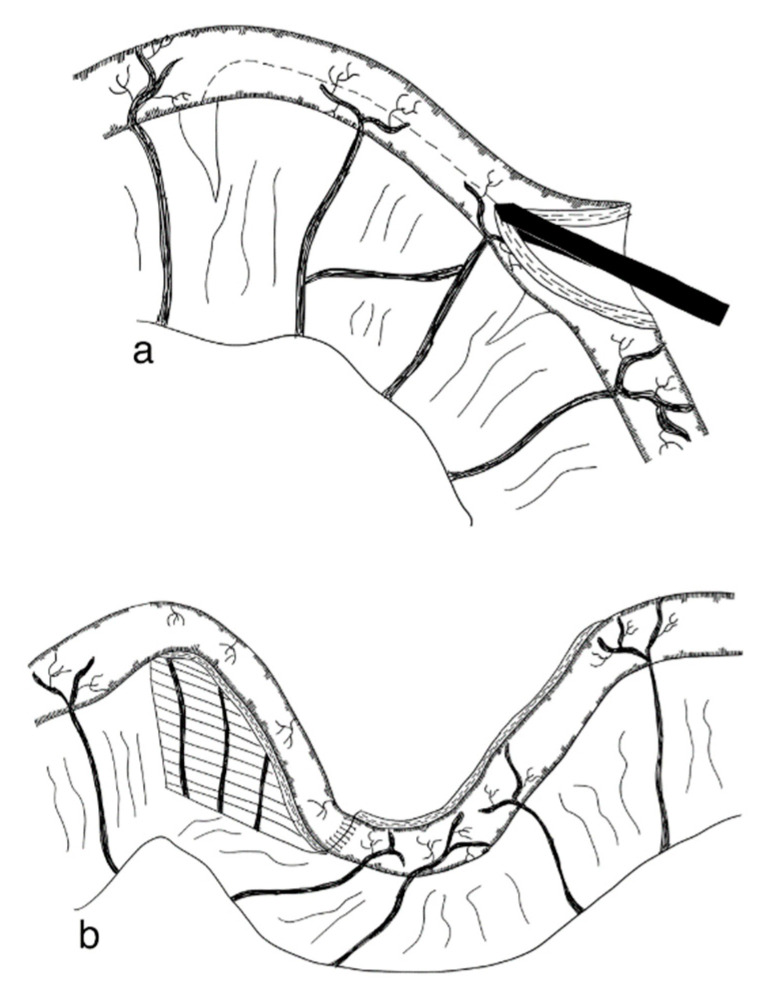
(**a**) Oblique and longitudinal division of the small intestine. (**b**) With a single anastomosis, intestinal continuity can be reestablished. Chahine and Ricketts [122].

This method was first described by Pokorny and Fowler in 1991 [106] and then applied in two patients by Chahine and Ricketts in 1998. This procedure is a modification of the LILT method, preventing the need for two longitudinal sutures and three end-to-end anastomoses [122]. The first part of this procedure is similar to the LILT method, separating the mesentery according to the bifurcated vessels. The small intestine is then divided with a stapler, first obliquely then longitudinally and ending obliquely on the other side. As a result, two divided segments, still connected to the remaining small intestine, can be created. With a single anastomosis, intestinal continuity can be re-established. Figure 4 exemplifies this surgical technique. This procedure has been applied in two patients; however, outcomes were not reported [122] (level of evidence: case reports).

#### 3.2.4. Double Barrel Enteroplasty (DBE)

**Figure 5:**
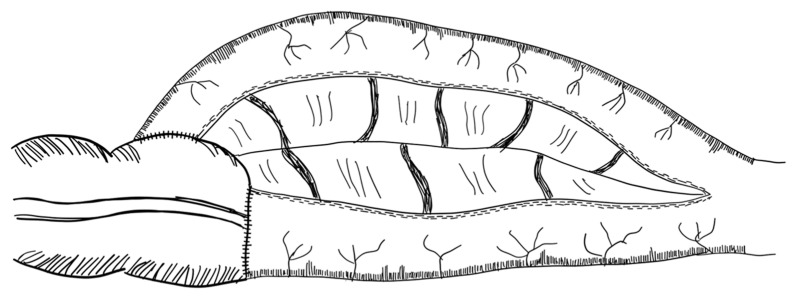
Dilated small intestine is longitudinally separated, creating two narrow tubes which are then reconnected to the colon. If the colon caliber is normal or small, one tube is anastomosed to the side of the colon. Shun et al. [123].

DBE is an adaptation of the original LILT technique. Similar to the LILT technique, parts of the small intestine that are dilated are longitudinally separated. In contrast to LILT, these two parts are not fashioned together into one narrow tube, but rather into two narrow tubes which run side by side. Through this, the traction on the mesenteric vessels and nerves is decreased (Figure 5). A retrospective cohort review of ten patients with SBS operated on with the DBE procedure between 2011 and 2018 found this technique a feasible and safe alternative to the original LILT technique, with 5/10 patients weaning from PN after a median follow-up of 39 months [123] (level of evidence: case series).

#### 3.2.5. Kimura Iowa Procedure

**Figure 6:**
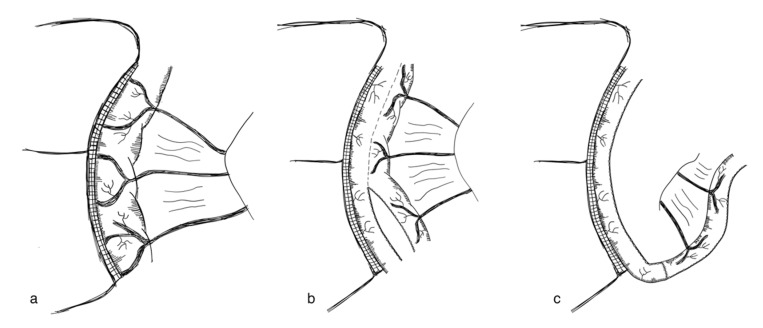
(**a**) Conduction of longitudinal seromyotomy on the antimesenteric side of the duodenum and exposing the submucosa. This part was then sutured to the liver and abdominal wall for neovascularization. (**b**) After eight weeks, the bowel segment was divided with a stapler into two loops (**c**) and end-to-end anastomosed to the remaining small intestine. Kimura and Soper [125].

The aim of Kimura and Soper was to develop a technique with which isolated bowel segments without mesenteric attachments could be created [124]. This technique could be used if the mesenteric blood supply is not mobile enough to perform, e.g., a LILT procedure. In 1990, Kimura and Soper reported on their first animal experiments [124]. In 1993, they reported on their first successful surgery of a six-week-old infant with a remaining small intestinal length of 17 cm due to intrauterine midgut volvulus after initial end-to-end duodeno-colostomy. In the first surgery a longitudinal seromyotomy on the antimesenteric side of the duodenum was conducted, exposing the submucosa. The capsule of the anterior liver margin and the peritoneum adjacent to the abdominal wall muscle were then removed, enabling them to suture the duodenal submucosa together. This created a segment of the duodenum with the original blood supply on the mesenteric side and a neo-blood supply on the antimesenteric side [125]. After eight weeks, the abdomen was re-entered for the lengthening procedure. However, the duodenum was partially detached from the liver and the abdominal wall, making a revision necessary. After another eight weeks, the duodenum, which was previously attached to the abdominal wall and the liver, was divided with a stapler into two loops and end-to-end anastomosed to the remaining small intestine. Immediately after the second operation, gastroesophageal reflux was diagnosed. This was treated with a fundoplication and a shunt duodeno-dunodenostomy. At the age of 18 months, he was able to absorb 50–60% of his daily caloric intake via the enteric route [125]. This procedure has not gained much interest, as it is very complex, requires at least two operations and delays a possible impact on absorption to the second procedure [75]. Figure 6 demonstrates the Kimura Iowa Procedure (level of evidence: case report).

#### 3.2.6. Spiral Intestinal Lengthening and Tailoring (SILT)

**Figure 7:**
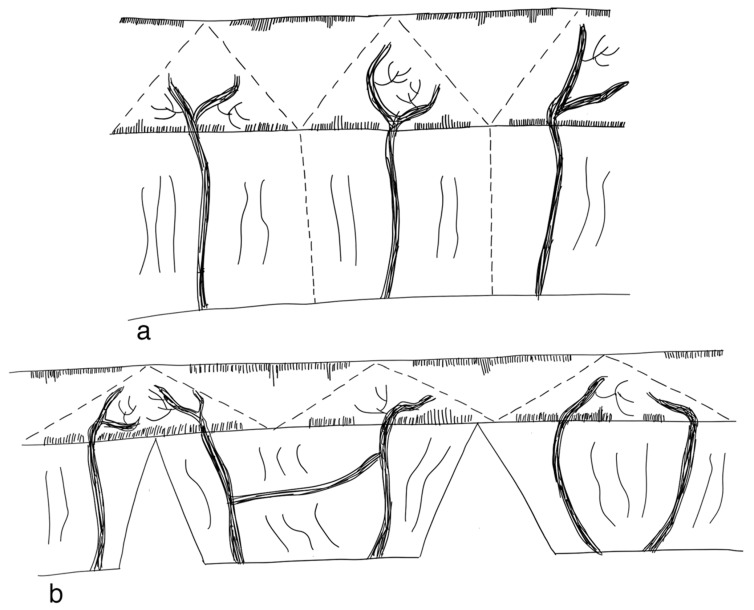
(**a**) The small bowel wall is cut spirally at 45° and 60° to its longitudinal axis. The mesentery is then incised where the previous spiral incision of the bowel met the mesenteric line. (**b**) The small bowel is then stretched longitudinally, twisted and anastomosed. Cserni et al. [132].

Spiral intestinal lengthening and tailoring (SILT) reflects a relatively new approach proposed in 2011 by Cserni et al. [126]. The small bowel wall is cut spirally at 45° and 60° to its longitudinal axis. The mesentery is then incised where the previous spiral incision of the bowel met the mesenteric line. The small bowel is then stretched longitudinally and twisted. The incision is closed with a continuous 4/0 vicryl suture [126] (Figure 7). Calculations have been conducted on how the small intestine should be cut in order to standardize this procedure [127]. 

Mehrabi et al. suggested a modification to the original SILT technique by leaving the mucosal layer intact. This is supposed to reduce the complication of postoperative intestinal leakage and abdominal abscess formation. It is described as technically and clinically feasible in animal models [128]. 

The SILT technique employs less manipulation of the mesentery compared to LILT and does not change the orientation of the muscle fibers like the STEP procedure [129]. Furthermore, staplers are not required, reducing the risk of bleeding. It can also be performed in the small intestine with less dilatation (≤4 cm) [82]. The SILT procedure has been successfully tested in animals [126,129]. In a case report, a ten-month-old boy with a remaining nine centimeters of jejunum was successfully operated on with the SILT technique. The small bowel segment had a 60% increase in length [130]. A study by Coletta et al. reported on five patients successfully operated on with the SILT technique. Median increase in small bowel length was 56%. No major complications related to SILT were observed and after a median follow-up of 26 months, no other surgical interventions had to be conducted [131]. It has been proposed that SILT could be performed as a standalone procedure or in combination with a STEP or LILT operation [130]. Evidence, however, is sparse and limited to a few cases (evidence level: case series).

#### 3.2.7. Transverse Flap Duodenoplasty (TFD)

**Figure 8:**
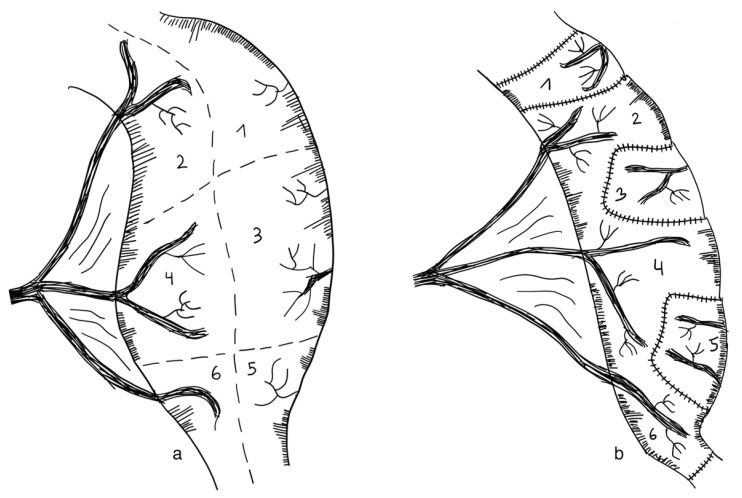
(**a**) The duodenum is cut longitudinally along the antimesenteric border. Three vascularized pedicle flaps are created, each with an anterior and posterior part. (**b**) These flaps are then spirally rotated and sutured together. The numbers 1–6 illustrate the movement of the different parts. Alberti et al. [133].

Most procedures for lengthening the short bowel are limited to the ileum and jejunum. Duodenal lengthening is more difficult, as blood supply is less tangible, and the pancreas, bile duct and major duodenal papilla are adjacent. Alberti et al. devised an approach for dilated duodenum in 2018. In a 2-month-old child with SBS, the duodenum was cut longitudinally along the antimesenteric border. Three vascularized pedicle flaps were created, each with an anterior and posterior part. These flaps were then spirally rotated and sutured together, lengthening and narrowing the duodenum [133] (Figure 8). These experiments are limited to one single case report to the best of our knowledge (level of evidence: case report).

#### 3.2.8. Composite Ileo-Colic Loop (CBT)

**Figure 9:**
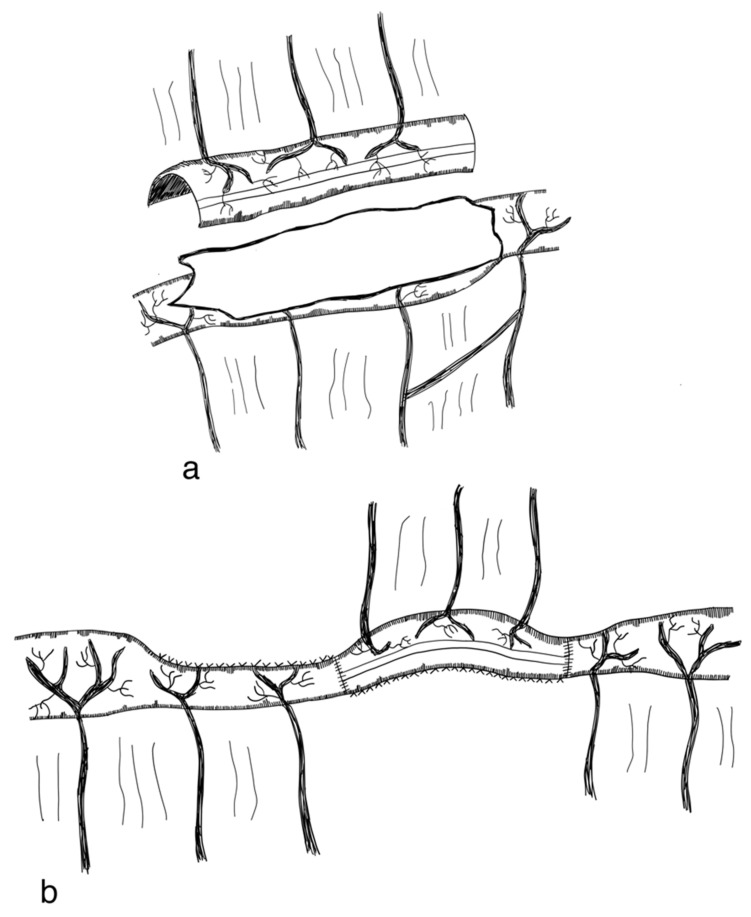
(**a**) Isolation of 30 cm colon segment, longitudinal cut along the antimesenteric border, and removal of colon mucosa. A 30 cm ileum segment was cut along the antimesenteric border. Isolated demucosed colon was then sutured to the exposed ileal submucosa. (**b**) Then, 8–10 weeks later, the modified 30 cm ileo-colic loop was cut in two halves, tubularized and anastomosed isoperistaltically to the remaining small intestine. Bianchi et al. [134].

A further step in the treatment of SBS was proposed by Bianchi, the inventor of the LILT procedure. In 1996, he proposed a two-step procedure, known as the composite ileo-colic loop or composite bowel tube (CBT). In a first operation, a 30 cm colon segment with its mesentery was isolated and cut on its antimesenteric border. The remaining colon was re-anastomosed. The mucosa of the colon segment was then removed at the submucosal plane. In the next step, a 30 cm ileum segment was cut along the antimesenteric border. The seromuscular layer was peeled away so the ileal mucosa was exposed. The previously isolated demucosed colon was then applied and sutured to the exposed ileal submucosa. Then, 8–10 weeks later, the modified 30 cm ileo-colic loop was cut in two halves, tubularized and anastomosed isoperistaltically to the remaining small intestine [134] (Figure 9). The original mesenteric side was further vascularized by the original ileal mesentery, while the antimesenteric side was vascularized by the colonic mesentery. CBT can be used alone or combined with other bowel lengthening techniques [134]. To our best of knowledge, these experiments have only been conducted in pigs (level of evidence: animal models).

#### 3.2.9. Mechanical Distraction

**Figure 10:**
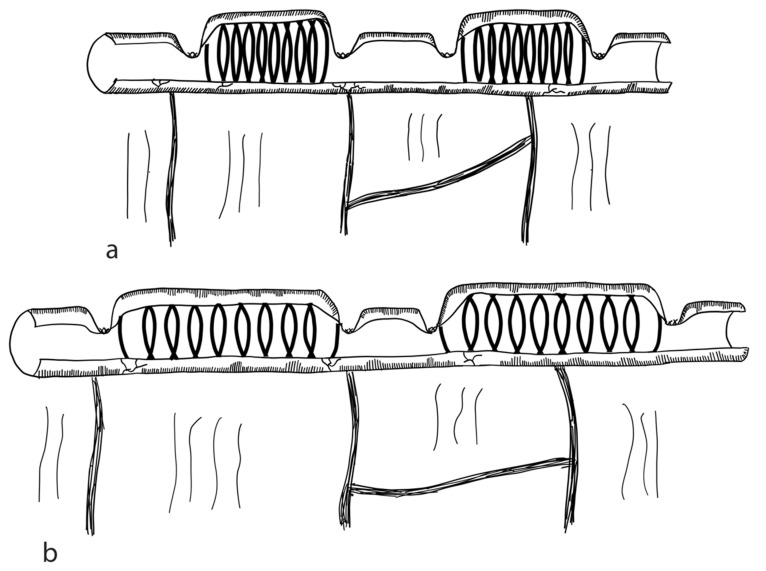
(**a**) Small intestine with introduced springs secured by plications. (**b**) Small intestine with elongated springs. Dubrovsky et al. [154].

Several research groups experiment on mechanical distraction of the small intestine. In 1997, Printz et al. reported a distraction apparatus in rabbits, achieving a nearly 10 cm increase in the length of the small intestine over three weeks [135]. Several devices were developed over the last two decades using different techniques, i.e., intestinal screws [136], hydraulic extenders [137,138], nitinol (nickel titanium) springs [139,140,141] or encapsulated polycaprolactone springs [142,143]. Dunn and collaborators showed small intestinal lengthening with a more than 3-fold increase in length following mechanical spring distraction [144]. In addition, a persistent increase in small intestinal diameter, thickening of the muscularis mucosae, hypertrophy of the villi, increased crypt depth and smooth muscle hypertrophy were observed, indicating that intestinal expansion was not simply the result of stretching, but rather intestinal growth had occurred [141,145,146]. Furthermore, the lengthened intestine showed peristalsis [147]. Finally, the lengthened intestine also demonstrated more macroscopic and microscopic blood vessels per length of mesentery compared to the control segments [148]. This group has also experimented with self-degrading 3D printed springs [149]. Nylon, a biocompatible material regularly used in sutures, was used as the printing material [150]. Further experiments explored the use of poly(caprolactone), also capable of self-degradation [142,143]. Their main aim is to develop a self-degrading spring that could be inserted endoscopically [149]. Figure 10 illustrates their surgical approach.

Moreover, Teitelbaum’s group has used balloon catheters for intestinal lengthening [151,152,153]. The lengthened segments were longer compared to control segments and had a 200% increase in surface area. Four weeks after re-implantation of the elongated small intestine, the length remained stable, and the motility of the small intestine was comparable to that of pig small intestine without any intervention. Some parameters (barrier function, mucosal disaccharidase levels and electrophysiological measurements) declined immediately after the elongation; however, they returned to normal levels after 28 days [137]. 

Many groups are experimenting with different techniques of mechanical distraction. However, as yet, no reported attempts have been made in humans, to our best of knowledge (level of evidence: animal models).

### 3.3. Procedures Slowing down Intestinal Transit without Bowel Lengthening

#### 3.3.1. Antiperistaltic Small Intestinal Segment

**Figure 11:**
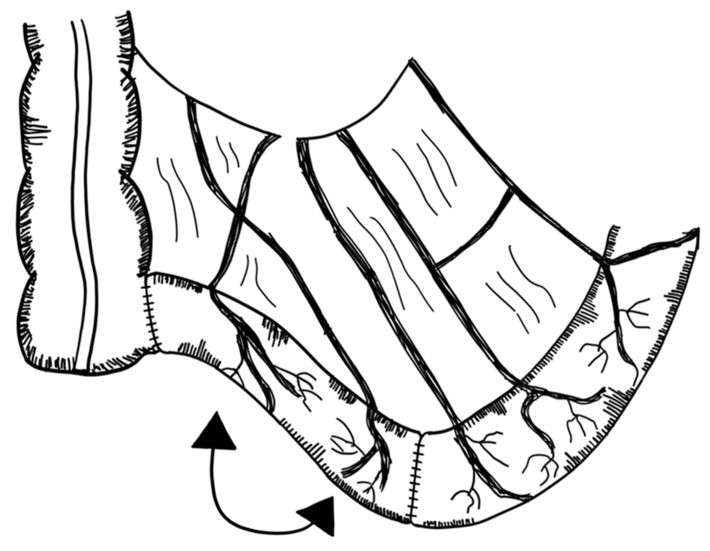
A segment of the small bowel is reversed (indicated by the arrow) and re-anastomosed with a jejuno-colic anastomosis. The reversed segment should measure about 3 cm in children, and it should be located in the distal part of the small bowel, in close proximity with the ileocecal valve. Beyer-Berjot et al. [158].

The first reported successful use of this technique dates back to 1962 and was performed in an 84 year old woman [155]. A small bowel segment is reversed and re-anastomosed with the small bowel in continuity [156,157,158,159], leading to an anti-peristaltic movement (Figure 11). Through this, the contact time of the nutrients with the intestinal epithelium can be increased. The reversed segment should hereby be located in the distal part of the small bowel, in close proximity to the ileocecal valve. The ideal length, long enough to slow down transit time without causing bowel obstruction, has been described as 3 cm in pediatric patients [101,102]. Several studies experimented with this technique; however, evidence is sparse, especially in pediatric patients (level of evidence: case reports).

#### 3.3.2. Colon Interposition

**Figure 12:**
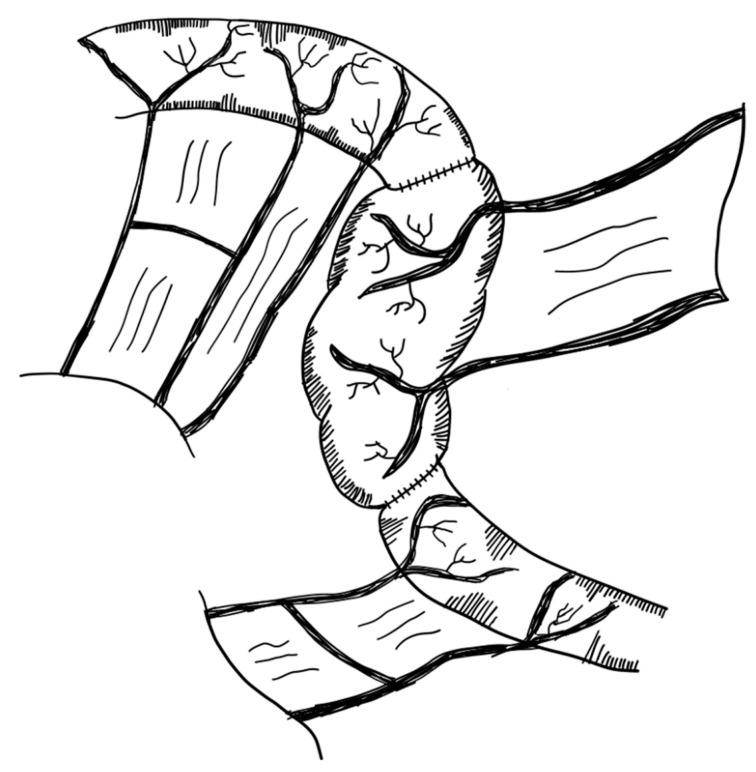
Interposition of a colon segment in the small intestine. Lloyd [160].

This technique comprises the interposition of a colon segment mostly between the jejunum and ileum [160,161] (Figure 12). The colon can be inserted in an isoperistaltic or an antiperistaltic direction, with the former having a better reported outcome [101,160,162,163]. The optimal length of the segment has been reported as 3 cm in pediatric patients. The idea is that the isoperistaltic colon prolongs the passing time of nutrients through the small intestine [101,164]. The interposed colon segment showed adaptive changes in animal models, taking over some small bowel features [161,165]. The main advantage of this method is that no small intestine is used [101]. This technique requires anastomoses. 

In 1984, six infants with SBS underwent isoperistaltic colon interposition. Three children were able to wean from parenteral nutrition with normal development after 84 months follow-up. The remaining three patients were not able to wean and died due to complications of the PN [166]. Evidence is limited to and relies on old case reports (level of evidence: case series).

#### 3.3.3. Intestinal Valves and Sphincters

**Figure 13:**
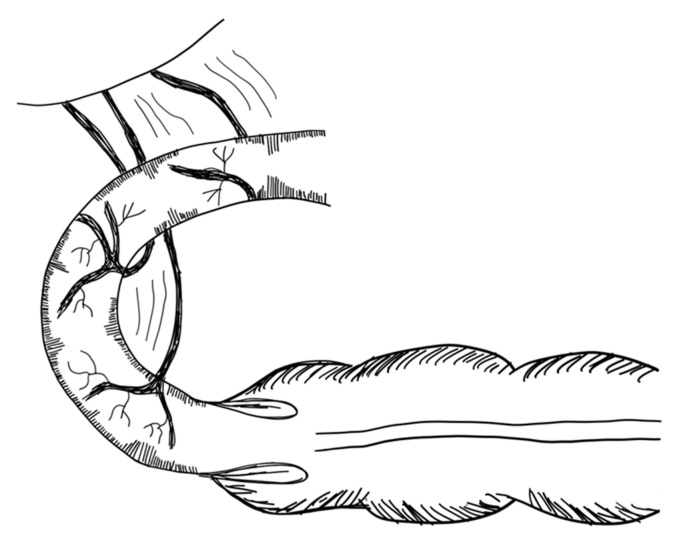
Nipple valve reconstruction. Shafiekhani et al. [169].

The idea of this approach is that a distally inserted valve or sphincter jams the proximal part of the small intestine, leading to an increased transit time. This also coincidentally dilates the proximal small intestine, enabling possible future LILT, STEP or other procedures [167]. Different types of valves have been described in the past [168]. A study by Shafiekhani et al. from 2021 reports on two cases where nipple valve reconstruction was performed in adults with acceptable outcomes [169] (Figure 13). To the best of our knowledge, no other studies report on the singular implantation of intestinal valves or sphincter (level of evidence: case reports).

#### 3.3.4. Recirculating Bowel Loops

**Figure 14:**
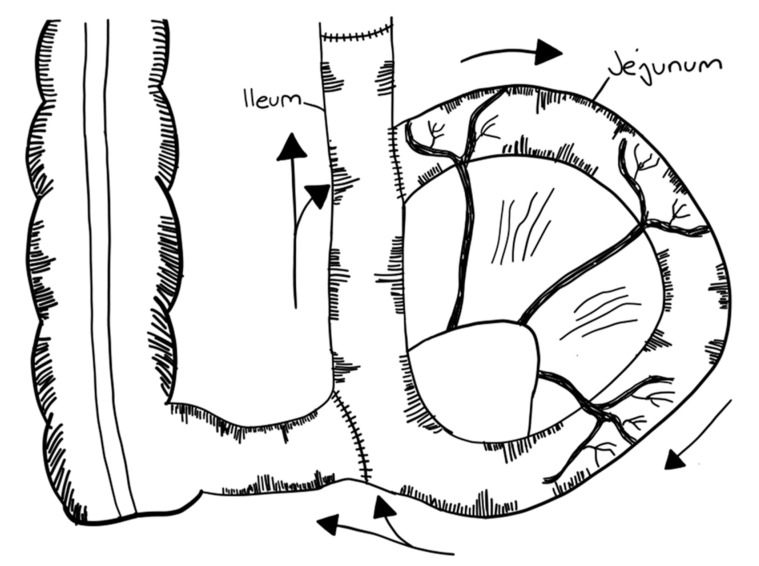
Reversal of parts of the small intestine and creation of an ileal loop. The arrows indicate the movement of the small intestinal content. Machby et al. [170].

In 1965, Mackby et al. experimented with different approaches to slow the transit time of nutrients passing through the small intestine. Their final approach was to combine a reversal of parts of the small intestine with an ileal loop formation (Figure 14). Hereby, part of the food intake circulates around this loop, prolonging the transit time. In combination with the reversed segment, this procedure produces a sufficient delay of the transit time. This operation was also successfully conducted in an adult woman, who survived for two years after the procedure [170]. In 1969, Poth reported on a similarly successful approach with a recirculation loop and a terminal antiperistaltic segment in a 34-year-old woman [171]. Furthermore, Camprodon et al. reported on a surgery in a young man with a favorable outcome after twelve months [172]. However, literature on reported recirculating bowel loops is sparse and is based on a few case reports of adult patients. Its efficacy is considered unproven [173] (level of evidence: case reports).

### 3.4. Procedures Improving Small Intestinal Motility without Bowel Lengthening

#### 3.4.1. Tailoring and Plication

This procedure may be applied to dilated portions of the intestine instead of the LILT or STEP procedures. In a dilated intestine, the surface to volume ratio is decreased and the peristalsis is less effective in propelling enteric contents forward. Reducing the diameter of the intestine may enhance absorption and improve motility without extensive division of the intestine. 

**Figure 15:**
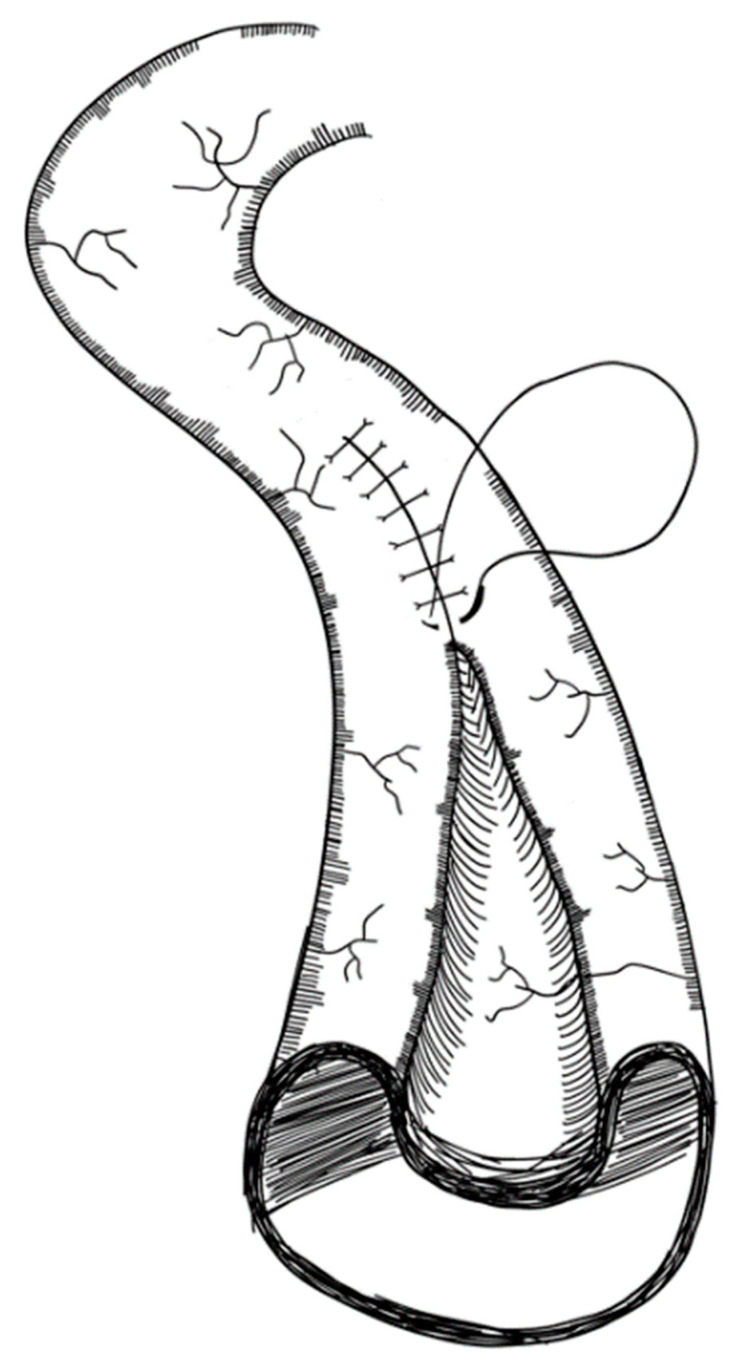
Plication of small intestine. de Lorimier and Harrison [176].

Bowel diameter is thereby reduced by resecting (tailoring) or imprinting (plication) of the small intestine on the antimesenteric side (Figure 15). Through tailoring, the absorptive surface area is decreased. With plication, mucosa can be preserved and motility is improved [174,175]. This technique can be applied to long lengths of the small intestine. Furthermore, the risk of leakage is minimal given that the integrity of the small intestine is left untouched. In 1983, de Lorimier and Harrison applied plication of the proximal dilated small bowel in twelve infants with jejunal atresia. Mucosa was retained, no obstructions were observed and most intestinal segments appeared normal [176]. Kimura et al. devised a similar approach in 1996 for patients with duodenal or jejunal atresia. A wide elliptical part of the antimesenteric seromuscular layer of the dilated small intestine is resected. The underlying submucosa and mucosa are left intact and are either inverted or imbricated into the small bowel. The muscular margins are then sutured together. This procedure was conducted on a five-year-old female patient with duodenal atresia. She already underwent surgical repair of the duodenal atresia after birth and was referred to the hospital due to failure to thrive. The previously described surgical procedure was performed on the dilated duodenum. After five years of follow-up, she was still thriving nutritionally, and a contrast study of the upper gastrointestinal tract four years after surgery showed no dilation of the duodenum [177] (level of evidence: case series).

#### 3.4.2. Modified Antimesenteric Tapering Enteroplasty

**Figure 16:**
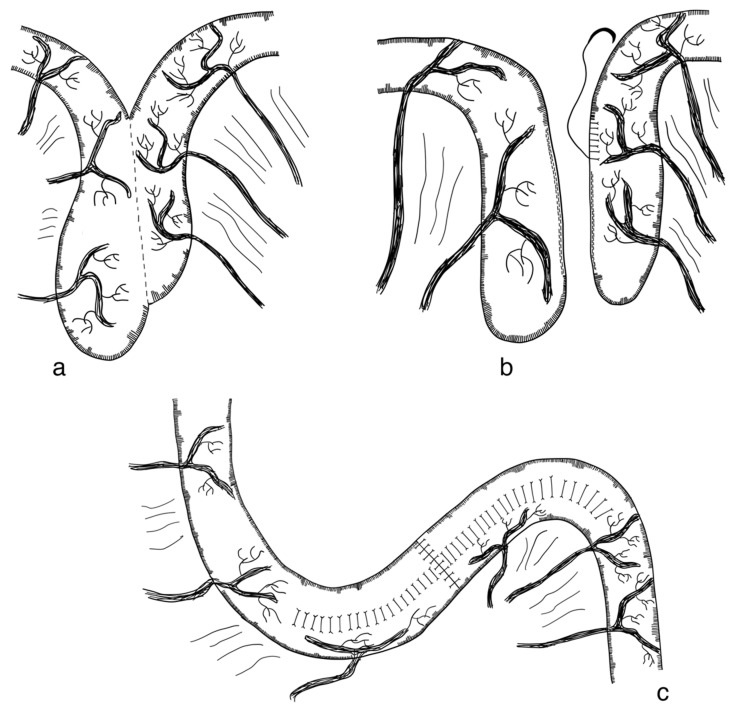
(**a**) Blunt division of the dilated side-to-side anastomosis. (**b**) Establishment of two blind loops of the small intestine, (**c**) which are then connected in an end-to-end isoperistaltic manner. Cruz [178].

This technique has been described as a bowel-sparing operation in patients with dysfunctional side-to-side anastomosis from a previous operation. The routine of this operation is to bluntly divide the mesentery leaves from both sides of the dilated side-to-side anastomosis. This anastomosis is then divided with a stapler and two blind loops of the small intestine are created, which are then connected in an end-to-end isoperistaltic manner (Figure 16). In 2021, Cruz reported on four adult patients, of which three were able to wean off PN [178] (level of evidence: case series).

### 3.5. Small Bowel Transplantation

The technical basis of intestinal transplantation (IT) was determined experimentally by Lillhei in 1959 and Starzl in 1960 using dogs [179,180]. Until the advent of tacrolimus in 1990, which significantly improved survival, IT was largely unsuccessful [181]. Only two cases of isolated IT, one of which was an infant, were reported to survive until 1990 [182,183]. Until recently, IT was still considered to be a high-risk operation and should only be performed as a last resort in specialized centers [38,184].

Indications for short bowel transplantation (SBT) are numerous; however, the grade of evidence is very low for any individual etiology. ESPEN lists the following: impending, progressive or overt liver failure due to intestinal-failure-associated liver disease (IFALD); thrombosis of two or more major venous vessels; two or more central line infections per year with sepsis; refractory electrolyte changes and frequent episodes of dehydration; high risk of death attributable to underlying disease such as congenital mucosal disorders; ultra-short bowel syndrome (gastrostomy, duodenostomy, residual small bowel <10 cm in infants and <20 cm in adults) and invasive intra-abdominal desmoid tumors; patients with high morbidity (frequent hospitalization, narcotic dependency, inability to function (i.e., pseudo-obstruction, high output stoma) or low acceptance of long-term PN, especially in young patients [74]. Furthermore, it has been stated that patients should be considered for IT with loss of venous access, sepsis secondary to infected thrombi, pulmonary embolism and alterations of growth and development in children [184,185].

Three main types of IT exist: the small bowel graft with or without the liver and a multivisceral transplantation including intestine, liver and stomach [186]. Most commonly used is an isolated small bowel graft [186]. For this donor, the jejunum and ileum were anastomosed to the recipient proximal jejunum. Often, a segment of donor colon is included given that the ileocecal valve seems to be associated with improved quality of life, continence and independence from PN [184,186,187]. Furthermore, the colon acts as a water absorber, as storage, and is active in the breakdown of food residues [187]. In patients with intestinal-failure-associated liver disease (IFALD), a combined liver and intestine transplantation is performed [184]. A preoperative liver biopsy is suggested to differentiate between reversible liver fibrosis and end-stage liver disease [188]. In the case of end-stage liver disease, a combined small intestinal and liver transplantation is necessary [107]. 

The intestinal transplant registry report from 2015 details the types of transplantation performed between the years 2001–2011. Small intestines without the liver were transplanted in 47% of patients, combined with the liver in 27% and multivisceral in 27% [186]. A newer analysis of the intestinal transplant registry from 2019 reports the most common type of transplantation to be small intestine plus liver (46%) followed by small intestine only (35%) [189]. Reported overall survival for patients which underwent any type of intestinal transplantation was 72.7% after one year and 57.2% after five years. Graft survival was 66.1% after one year and 47.8% after five years, respectively [189] (level of evidence: case series). 

Table 2 gives an overview over the different treatment approaches.

### 3.6. Tissue Engineered Small Intestines (TESI)

Shortage of donor tissue and high rejection rates of human allografts, alongside the need for life-long immunosuppression to tolerate the grafted tissue, mean new strategies are required to provide less/non-immunogenic transplantable tissues for patients with severe forms of SBS [190,191], in particular for patients with less than 40 cm of functional small bowel (and no ICV) for whom operative adaptation techniques are generally not applicable [192].

The most promising solution lies with tissue engineered small intestines (TESI). TESI is used to describe a number of different methods to biomedically engineer functional small intestinal tissue for graft or transplant. They generally consist of both cellular material and a (synthetic or natural) scaffold [193], although scaffold-free constructs are also under investigation [194]. The benefit of tissue engineered grafts is the ability to tailor the graft to the specific needs of the patient. Thus, patients with extensive intestinal resection would require full-thickness grafts, while for others grafting of the mucosa alone, for example, may be sufficient to improve intestinal absorption [191]. Some discussion exists as to what constitutes a full-thickness small intestinal graft, but it is widely accepted that a highly vascularized, innervated smooth muscle layer with an absorptive mucosa is essential [193,195]. Some authors argue that a lymphatic network and microbiota should also be included in the engineered tissue, due to their important role in intestinal immune function [196], but this is not widely explored [193,195]. Additionally, while most of the attention has been paid to the mucosa, the bioengineering of a functional muscularis will be needed as well. Much research with regards to TESI is underway and several clear strategies have emerged: small intestine submucosal grafts (SIS), stem-cell-derived and organoid-derived constructs. 

In the case of stem cells and organoids, both are grown on scaffolds in vivo or in vitro, which provide support and ease of handling. A promising line of research is the use of organoids or stem cells for intestinal repurposing, often referred to as small intestinalized colon (SIC). There are many similarities between SIC and SIS vs. other stem cell and organoid strategies, the main one being that SIC and SIS rely on decellularized donor scaffolds (with or without retaining vasculature). However, in SIS, the growth of cellular networks on the scaffold is mediated by diffusion of cells from neighboring healthy tissue in vivo, while for SIC, stem cells or organoids are generally cultured in vitro before seeding onto the repurposed tissue scaffold. Non-SIC stem cells and organoid strategies rely on other natural or synthetic polymer scaffolds to support tissue formation, with seeding and culture performed primarily in vitro, at least initially, before transplantation into an animal model. 

While no TESI approach has been trialed in human patients to date, SIC and its reliance on a pre-existing fully vascularized but decellularized intestinal or colonic tissue scaffold is likely the closest to application in clinics. Both stem cell and organoid techniques are extraordinarily promising and may well provide a cure for SBS in the future once challenges related to graft size and vascularization have been fully remedied. An overview of these strategies is provided in the following sections.

#### 3.6.1. Small Intestinal Submucosa (SIS) Grafts

Small intestinal submucosa (SIS) is an acellular, collagen-based bioscaffold, widely used in tissue repair, e.g., in skin, myocardium, blood vessels, bone, tendons and in the gastrointestinal tract [197,198,199,200,201]. SIS is fabricated by extracting the submucosal layer of intestine, often porcine-derived, which is then decellularized [197,202,203,204]. This can be retained as a bioscaffold sheet or solubilized to prepare extracellular matrix (ECM) hydrogels [205] which can be seeded with cellular material for graft construction. Cell-laden ECM hydrogels can then be injected directly into a variety of different organs as patches allowing tissue to be cultured in situ [206]. Initial studies suggest that neointestine based on SIS has the potential to develop structural features of the normal intestine, allows rapid regeneration of the mucosa and the smooth muscle fibers and contains functional growth factors [200,201,207]. A study from 2001 conducted with xenografts showed that SIS patches can be used for increasing the total absorptive surface area, however, tubular replacement of the small bowel with SIS was not feasible [199]. A major limitation of the SIS method is the extent of vascularization of the graft. Effective and rapid vascularization is necessary to ensure nutrient and oxygen supplies to the graft tissue and prevent damage [208]. Nowocin and co-workers proposed a method of decellularizing a segment of porcine ileum while maintaining an intact vasculature and partial retention of the submucosa, muscularis and serosa [209], allowing immediate perfusion of the scaffold with the host’s blood post-implantation. Such strategies significantly improve the chance of graft success in the longer term; however, no attempts were made to examine the extent to which other cells can recolonize the scaffold in vivo in this work. In 2015, Nakao et al. presented a novel surgical approach consisting of dividing the small bowel as in the LILT method. However, instead of sewing the two parts together, they were connected with the SIS graft. These two parts are then reconnected with the rest of the small intestine similar to the LILT method. Using this method, the total absorptive surface area can theoretically increase by 100% [202] (level of evidence: animal models).

#### 3.6.2. Stem Cells

Stem-cell-based TESI are constructs engineered from intestinal stem cells (ISCs) or induced pluripotent stem cells (iPSCs) cultured on a scaffold. These scaffolds can be prepared by electrospinning, 3D printing or molding. After cells have been cultured on and within the scaffold, they are used as patches or rolled to create a tubular construct. The porosity, organization and mechanical strength of the scaffolds (decellularized, natural polymer or synthetic polymer) are crucial to the overall success of the construct, as they aid the differentiation of ISCs and iPSCs and their assembly into organized networks that become the musculature, vasculature, innervation or mucosa of the small intestine.

Decellularized scaffolds composed of natural ECM were mentioned in the previous section. Their primary advantage is their low or non-immunogenicity (depending on the source) and rapid vascularization due to the presence of proteins and growth factors in their composition. However, they suffer from low availability and low batch-to-batch reproducibility. Natural polymer scaffolds, primarily chitosan and silk fibroin, are also highly biocompatible and are available in large quantities but often suffer from issues of mechanical stability, which affect their ability to withstand typical pressures observed in the intestines. Synthetic polymer scaffolds are most commonly based on poly(glycolic acid) (PGA), poly(lactic-co-glycolic acid) (PLGA) or poly(caprolactone) (PCL). They have the advantage of being highly modifiable, resulting in very defined mechanical properties and organization, highly important for differentiation and final applications in vivo. However, they are often less biocompatible than natural materials and degrade over a longer timeframe, which can result in cell damage due to immune response.

Once the scaffold has been selected and prepared, cells are seeded onto the support and cultured in vitro for several weeks before implantation to allow a confluent density of cells both on and through the scaffold, creating a 3D tissue structure that will ideally be maintained once the scaffold has degraded. Some recent successful examples of stem-cell-based TESI have exploited chitosan/collagen [210] composites, PCL [211,212] and silk fibroin [213] as the scaffold.

For example, Zakhem et al. created an innervated smooth muscle on a chitosan/collagen composite scaffold [210]. First, smooth muscle cells (SMCs) were seeded on a wavy, laminin-coated polydimethylsiloxane (PDMS) mold for culturing. After 5 days, a collagen gel containing neural progenitor cells was applied over the SMCs and cultured for a further 15 days [214] to create an innervated smooth muscle layer. This construct was removed from the wavy PDMS form and wrapped onto a freeze-dried chitosan/collagen scaffold for implantation. Constructs were implanted in the subcutaneous pockets of athymic rats and analyzed after 14 days in vivo. These constructs were found to be vascularized within the 14-day implantation, they exhibited luminal patency, the contractile phenotype of the smooth muscle tissue was maintained and the smooth muscle and nerve cells were also highly organized within the tissue construct. However, contractile motion in the innervated tissue was lower than that in real tissue, indicating that the innervation of the tissue may have not been complete within the time window chosen. Further experiments must also be conducted to examine the potential for inclusion of an epithelial layer, as well as the effect of orthotopic implantation.

Chen and co-workers also successfully created a highly organized and aligned innervated smooth muscle layer on a freeze-cast silk fibroin scaffold [213]. Two aligned silk scaffolds were seeded with SMCs and rolled around an internal support to create the tubular structure of the intestines, with the internal scaffold aligned to represent circular smooth muscle and the outer to represent longitudinal smooth muscle. The seeded and rolled scaffold was cultured for 3 days before unrolling to seed neural stem cells. The scaffold was reformed and allowed to culture for 2 weeks to create innervated smooth muscle. These constructs were cultured entirely in vitro, and no implantation was attempted, thus no vascularization of the tissue was observed. However, neurite outgrowth was detected in the cultured tissue, suggesting innervation of the tissue. Furthermore, markers of smooth muscle differentiation were also detected, specifically markers related to contractile motion. Despite this, no contractile motion of the tissue could be measured under stimulus.

No or weak contractile motion of smooth muscle tissue is a challenge in engineering graft tissue full-thickness TESI from stem cells [193,195]. Many authors have outlined the importance of a dynamic culture environment in ensuring the correct functioning of the smooth muscle layer [191,215] in the GI tract. Dunn and co-workers have put forward two promising strategies to ensure maintenance of the contractile function of SMCs during culture [211,212]. The first is the use of a specially tailored culture medium that promotes the proliferation of neural cells and interstitial cells of Cajal (ICCs) alongside SMCs, ensuring innervation of the growing muscle layer [212]. More recently, they demonstrated co-culture of SMCs and ICCs on STO fibroblast-seeded PCL scaffolds [211]. The scaffolds were electrospun and aligned to ensure correct organization of the cells during culture, and this approach demonstrated that cultured SMCs maintained normal contractile function for up to 10 weeks. Coupling these strategies with previously described examples could be key to ensuring correct function of the smooth muscle layer in stem-cell-based TESI (level of evidence: animal models).

#### 3.6.3. Organoid Units

While stem-cell-based TESIs are making great strides, by far the most advanced TESI strategy is organoid-based constructs. Organoid units are three-dimensional structures built in vitro by different cells resembling their organ of origin [216]. They have been employed extensively in in vitro experiments, to study physiological responses in their organ of origin, primarily in drug discovery and toxicology studies [216]. However, they can also be cultured in vitro on a suitable scaffold to create larger sections of their organ of origin suitable for transplant or graft [217]. Organoids of the small intestine can be derived from a single biopsy of epithelium [218]. A study by Grant et al. from 2015 uses mouse or human donor intestines to generate organoid units, which were then implanted in genetically identical or immunodeficient host mice. After 4 weeks, the tissue-engineered small intestine (TESI) was extracted and used for further analyses. The reported TESI had a well-differentiated epithelium with intact ion channels, tight junctions, microvilli and functional brush border enzymes [219]. More recently, Meran et al. used pediatric patient biomaterial derived from two endoscopic epithelial biopsies of 12 patients with intestinal failure or those at risk for intestinal failure to create jejunal organoids [191]. After 8 weeks of in vitro culture, the jejunal organoids were seeded onto either decellularized human small intestinal or colon scaffolds, creating patient-derived engineered jejunal grafts that were then implanted into mice and pig models. Examination of the grafted tissue after removal identified macroscopic luminal structures throughout several of the grafts, indicative of neovascularization. Stromal infiltration of the graft, as well as goblet cell and enterocyte differentiation, were highly dependent on the location of the implant in the model. Furthermore, both small intestine and colon scaffolds showed no significant differences in graft formation, leading to more potential opportunities for utilizing a patient’s own existing intestinal tissue to serve as scaffolds for this technique. Orthotopic transplants, eventually in human patients, as well as strategies to improve vascularization over a shorter time period [209,220,221], will be required to fully assess the success of these tissue engineering jejunal grafts. However, as a proof-of-concept, using patient-derived cellular material seeded into human donor scaffolds, great strides have been made towards individualized intestinal reconstruction using organoids units [191]. Sugimoto et al. addressed the issue of orthotopic implantation in a more recent work, in which human and rat ileal organoids were seeded into demucosed rat colons and implanted at the ileocecal junction in both mice and rat models [218]. Implantation in the intestinal tract ensured that the construct was exposed to the normal intestinal environment. Analysis of the implanted construct showed that the organoid-derived SIC had absorptive function, vasculature, innervation, villi, lymphatics and greatly improved intestinal failure in SBS rat models, bringing organoid-based TESI even closer to clinical applications. However, it must be noted that the authors reported gut microbiota changes after implantation, which requires more investigation before human applications can be considered [218,222] (level of evidence: animal models).

## 4. Conclusions

In conclusion, despite major advances in the state of the art across all fronts (conservative, surgical and regenerative treatments), SBS remains a devastating diagnosis with no definitive cure.

Conservative approaches remain the front line of treatment for patients with short bowel syndrome. Parenteral nutrition is a crucial cornerstone of conservative treatment in the first line, especially in the initial phase of disease. This is accompanied by antibiotics, PPIs or H2-blockers, antimotility agents, bile-acid binding resins and pancreatic enzyme replacement in order to address clinically significant consequences of SBS such as small intestinal bacterial overgrowth, gastric hypersecretion, diarrhea and fat malabsorption. Chyme reinfusion has seen a renaissance and is recommended by the ASPEN and ESPEN wherever possible in patients with double enterostomies or entero-atmospheric fistulas. The greatest advancement in recent years has been the establishment of the glucagon-like-peptide 2 (GLP-2) analogue teduglutide to increase intestinal absorption. This has been shown to be safe and effective and enables reduction of parenteral support in the majority of cases. and even enteral autonomy in some cases [66]. 

However, although conservative treatments for SBS are essential for patient care, the primary focus of this review has been to highlight the major surgical advances towards curative treatments for SBS. In the past six decades several promising surgical techniques have emerged, advancing the state of the art in SBS management, bringing us closer to a definitive cure. The LILT and STEP procedures, discussed above, are the most performed methods today [82] and have spawned new approaches with the aim of refining surgical management further still. Nevertheless, a cure for short bowel syndrome remains elusive. However, the future remains bright. New surgical methods under development, e.g., the implantation of self-expanding springs can potentially lead to a less invasive treatment of SBS by an endoscopic intervention [149]. Furthermore, the field of regenerative medicine is progressing at an extraordinary pace, opening up the possibility of repairing and replacing intestinal tissue on demand. A variety of tissue engineered small intestines (TESI), as they are collectively known, have been developed in the last years. TESI approaches range from small intestinal submucosa grafts to intestinal tissue repurposing with either stem cells or organoid units, the latter of which appears extraordinarily promising. Preliminary studies have shown great improvement in SBS animal models [218,222], making the regeneration of small intestinal tissue from a patient’s own tissues a distinct possibility in the not-too-distant future. The combination of the TESI approach with surgical interventions is another powerful tool with the potential to improve the quality of life and outcomes of patients with SBS. For example, the LILT procedure in combination with small intestinal submucosal (SIS) grafts has been proposed and tested in dog models with positive outcomes [202]. 

With the significant improvement in the state of the art of surgical interventions, alongside rapid developments in the field of regenerative medicine in the past decade, the overall confidence in the field is high that a cure for SBS is within reach. However, the path to a cure is not smooth, and more time and energy needs to be invested to safely transfer these new research approaches from animal models into a clinical environment.

## Figures and Tables

**Table 1:** Overview of causes of small bowel syndrome in children and adults. Adapted from Buchman and DiBaise et al. [11,13].

	Children	Adults
Congenital	Jejunal atresia Ileal atresia	-
Acquired (extensive small bowel resection)	Necrotizing enterocolitis Malrotation with midgut volvulus Gastroschisis Extensive aganglionosis Trauma	Crohn’s disease Catastrophic mesenteric events (arterial embolism, venous thrombosis) Volvulus Trauma Adhesive obstruction Malignancies Radiation enteritis
Functional SBS in severe malabsorption (bowel length intact)	Chronic intestinal pseudo-obstruction syndrome Refractory sprue Radiation enteritis Congenital villous atrophy	

**Table 2:** Overview over the different treatment approaches.

Method	First Description (Year)	Advantages	Disadvantages	Technical Difficulty *	Human Models	Success Rate	Evidence
**Procedures increasing small intestinal length**							
Serial transverse enteroplasty procedure	2003 [79]	Technically easy, reSTEP possible	Bleeding from staples might be difficult to control	+	Several	45% PN weanings	Case series
Longitudinal intestinal lengthening and tailoring procedure	1980 [99]	A lot of experience, avoidance of staplers	Manipulation of mesentery, three anastomoses, technically demanding, no second LILT after primary LILT possible	+++	Several	169 of 324 (52%) weanings from PN	Case series
Modification of LILTs with one anastomosis	1991 [106]	Only one anastomosis needed	Manipulation of mesentery, technically demanding, no second LILT after primary LILT possible, lack of experience	+++	Two patients with no reported outcomes	Unknown	Case reports
Double barrel enteroplasty	2008 [123]	Less traction on the mesenteric vessels than LILT	Manipulation of mesentery, technically demanding, no second LILT after primary LILT possible, lack of experience	+++	Ten patients from a single institution	5/10 weanings after 39 months, no deaths	Case series
Kimura Iowa procedure	1990 [124]	Possible if mesenteric blood supply is not mobile enough for other approaches	Technically demanding, requires at least two operations, lack of experience	++++	One patient	50–60% of daily caloric intake via the enteric route after 18 months	Case report
Spiral intestinal lengthening and tailoring	2011 [126]	Less mesenteric manipulation of mesentery than LILT, only limited bowel dilation necessary	Mesenteric manipulation, long anastomosis, limited lengthening, low evidence	+++	<10 cases	No major complications after a median follow-up of 26 months	Case series
Transverse flap duodenoplasty	2018 [133]	Possible for duodenum	Limited lengthening, low evidence, technically demanding, possible injury to Ampulla of Vater	+++	One 2-month-old child	Enteral feeding accounting for 54% of caloric intake	Case report
Composite ileo-colic loop	1996 [134]	Enables LILT procedure in non-dilated small bowel	Technically demanding, requires at least two operations, lack of experience, colon needs to be present	++++	Only pig models	/	Animal models
Mechanical distraction	1997 [135]	Increases length and surface area without the need for dilated bowel	Use of foreign material, one- or two-stage procedure, spring may obstruct or perforate small bowel	++	Pig and rodent models	/	Animal models
Small bowel transplantation	1959 [179]	Last choice, does not depend on bowel length/dilation	Immunosupression, high mortality, technically demanding	++++	Several	Patient survival rates were reported as 76%, 56% and 43% at 1, 5 and 10 years	Case series
**Procedures slowing down intestinal transit without bowel lengthening**							
Antiperistaltic small intestinal segment	1962 [155]	Increases transit time, technically easy	Small effect, low evidence	+	Several	38 adult patients, 17 weanings (45%), deaths 16% after 5 years follow-up	Case series
Colon interposition	1984 [166]	Technically easy, may be used in cases with very short, small bowel	Colon needs to be present, low evidence, three anastomoses	+	Six infants	3/6 weaning from PN	Case series
Intestinal valves and sphincters	1994 [167]	Dilation of small intestine with possibility to perform LILT/STEP/other lengthening procedures later	Intestinal stasis and bacterial overgrowth, low evidence, possible intestinal obstruction	+	Few cases	Acceptable surgical outcome	Case reports
Recirculating bowel loops	1965 [170]	Increases transit time, technically easy	Small effect, low evidence, intestinal stasis and bacterial overgrowth	++	Three adult cases	Outcomes were reported as successful and favor- able	Case reports
**Procedures improving small intestinal motility without bowel lengthening**							
Tailoring and plication	1983 [176]	Improvement of motility, technically easy	Low evidence, needs to be weighed against lengthening procedures (LILT, STEP, etc.)	+	Twelve infants	Mucosa was retained, no obstructions were observed and most intestinal segments appeared normal	Case series
Modified antimesenteric tapering enteroplasty	2021 [178]	Reduces stasis, technically easy	long anastomosis, low evidence	+	In four adult patients	3/4 were able to wean of PN	Case series

* Increasing difficulty from + to ++++, LILT = longitudinal intestinal lengthening and tailoring procedure, STEP = serial transverse enteroplasty procedure.

## Data Availability

All data generated or analyzed during this study are included in this published article.
